# Heterogeneity of paclitaxel distribution in different tumor models assessed by MALDI mass spectrometry imaging

**DOI:** 10.1038/srep39284

**Published:** 2016-12-21

**Authors:** Silvia Giordano, Massimo Zucchetti, Alessandra Decio, Marta Cesca, Ilaria Fuso Nerini, Marika Maiezza, Mariella Ferrari, Simonetta Andrea Licandro, Roberta Frapolli, Raffaella Giavazzi, D’Incalci Maurizio, Enrico Davoli, Lavinia Morosi

**Affiliations:** 1 Mass Spectrometry Laboratory, IRCCS Istituto di Ricerche Farmacologiche Mario Negri, Via La Masa, 19 – 20156 Milan, Italy; 2 Department of Oncology, IRCCS Istituto di Ricerche Farmacologiche Mario Negri, Via La Masa, 19 – 20156 Milan, Italy

## Abstract

The penetration of anticancer drugs in solid tumors is important to ensure the therapeutic effect, so methods are needed to understand drug distribution in different parts of the tumor. Mass spectrometry imaging (MSI) has great potential in this field to visualize drug distribution in organs and tumor tissues with good spatial resolution and superior specificity. We present an accurate and reproducible imaging method to investigate the variation of drug distribution in different parts of solid tumors. The method was applied to study the distribution of paclitaxel in three ovarian cancer models with different histopathological characteristics and in colon cancer (HCT116), breast cancer (MDA-MB-231) and malignant pleural mesothelioma (MPM487). The heterogeneous drug penetration in the tumors is evident from the MALDI imaging results and from the images analysis. The differences between the various models do not always relate to significant changes in drug content in tumor homogenate examined by classical HPLC analysis. The specificity of the method clarifies the heterogeneity of the drug distribution that is analyzed from a quantitative point of view too, highlighting how marked are the variations of paclitaxel amounts in different part of solid tumors.

Pharmacokinetic studies on anticancer drugs have been usually performed using HPLC or LC-MS/MS, measuring the drug concentration in plasma in order to define classical pharmacokinetic parameters. It is assumed that the drug concentration in plasma is in equilibrium with the concentration in tissue^1^. This is true for normal tissues in physiological conditions, but it does not hold for the drug concentration in the tumor tissues, where the distribution varies widely. Drugs can be measured in tumor homogenate with analytical techniques that are sufficiently sensitive and selective. However this method provides a measure of total drug concentrations that does not give a picture of the heterogeneity of drug distribution in tissues, related to the structural complexity of the neoplastic tissue and the influence of tumor microenvironment that markedly affect drug penetration^2^. Therefore methods are needed to show the drug’s spatial distribution in the tumor tissue^3^. The penetration of a drug in tumor depends first on its physicochemical characteristics, but the abnormal vasculature, reactive stroma, and inflammation that characterize tumor microenvironment are all important^4^. Since complete and efficient penetration of anticancer drugs in solid tumors is essential for optimal therapeutic effect, techniques that show how the drug distributes inside the tumor architecture would certainly help in understanding one of the causes of inefficient responses to chemotherapy or drug resistance^5^.

Mass spectrometry imaging (MSI) is a novel and interesting technique which increases the number of tools available to obtain information on the spatial distribution of a drug^6^. In comparison with other imaging techniques such as fluorescence microscopy, positron emission tomography, magnetic resonance spectroscopy and autoradiography, it can visualize drug distribution in organs and tumor tissues with good spatial resolution and better specificity, detecting the parent compound and metabolites simultaneously in a single experiment, without having to label the analyte^7^. Besides, MSI can analyze at the same time, endogenous compounds of interest co-localizing drugs with particular structure of the tissue or biological markers^8^. Another important advantage of MSI is that the tissue is not destroyed during measurement, so mass spectrometry can be combined with histological information^9^. This approach has even some limitation: it is only applicable to molecules that are ionizable by the MALDI or SIMS process, moreover the interpretation of quantitative data is often complex especially because of ion suppression effect and the chemical noise from the matrix ions covering the drug ion signal and finally, the sensitivity is quite limited if compared to autoradiography^10^.

We recently developed a MALDI MSI method using TiO_2_ nanoparticles as the matrix with resolution between 20 and 100 μm to visualize the spatial distribution of the well known anticancer agent, paclitaxel, in thin slices of tumor tissue^11^. We were able to detect paclitaxel in normal and tumor tissue, and its spatial distribution in tumor provides information on whether pharmacological responses are related to inadequate drug penetration, for instance in poorly vascularized parts of the tumor^12^. The use of a suspension of commercially available TiO_2_ nanoparticles as MALDI matrix resulted suitable for small molecule imaging because of the uniform nanoparticles deposition over tissues and the lack of background signals from matrix degradation^13^.

The MALDI MSI analysis of heterogeneous tissues such as tumors is challenging mostly because of the ion suppression effect that may be different in the various parts of the tumor section. The ion suppression effect is mainly due to endogenous species (e.g. lipids) competing for ionization or to salts accumulation typical of biological tissue^8^. Two different methods have been proposed to take into account the ion suppression effect. (i) The calculation approach: the analyte is applied on the sample surface and the image is acquired in order to calculate the suppression factors for each area or tissue of interest. The factors calculated are used to normalize the ion signal^14^. (ii) The normalization approach: the analyte ion signal is normalized using the total ion current^15^ or a reference peak present in the tissue or added in the matrix^16^.

We chose the latter method, based on a reference peak corresponding to deuterated analyte standard, uniformly applied over the tissue, to compensate ion suppression and laser shot-to-shot variability^17^. The optimized method presented here, was highly reproducible allowing us to investigate the distribution of paclitaxel in different models of human tumors implanted in nude mice. We examined tumors of different origin (ovary, colon, pleura and breast) with different sensitivity to paclitaxel. The data were combined with histological analysis of the same samples in order to associate areas of the tumor with the presence of the drug and areas of the tissues with distinct morphology.

## Results and Discussion

### Method set-up

**Figure 1:**
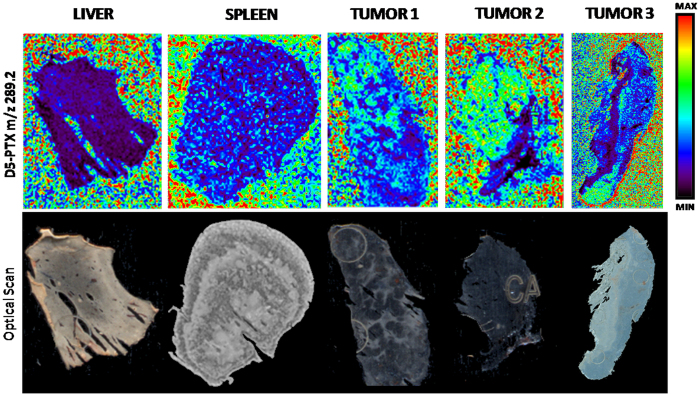
Ion suppression effect on the d5-PTX ion signal (m/z 289.2) of different tissues. Deuterated internal standard was dissolved in NP-TiO_2_ solution at the concentration of 3 μg/mL and sprayed onto the MALDI plate. The suppression effect differs between tissues, and even inside the same tumor section due to tissue heterogeneity. Upper panel, MSI images; lower panel, corresponding optical scans.

**Figure 2:**
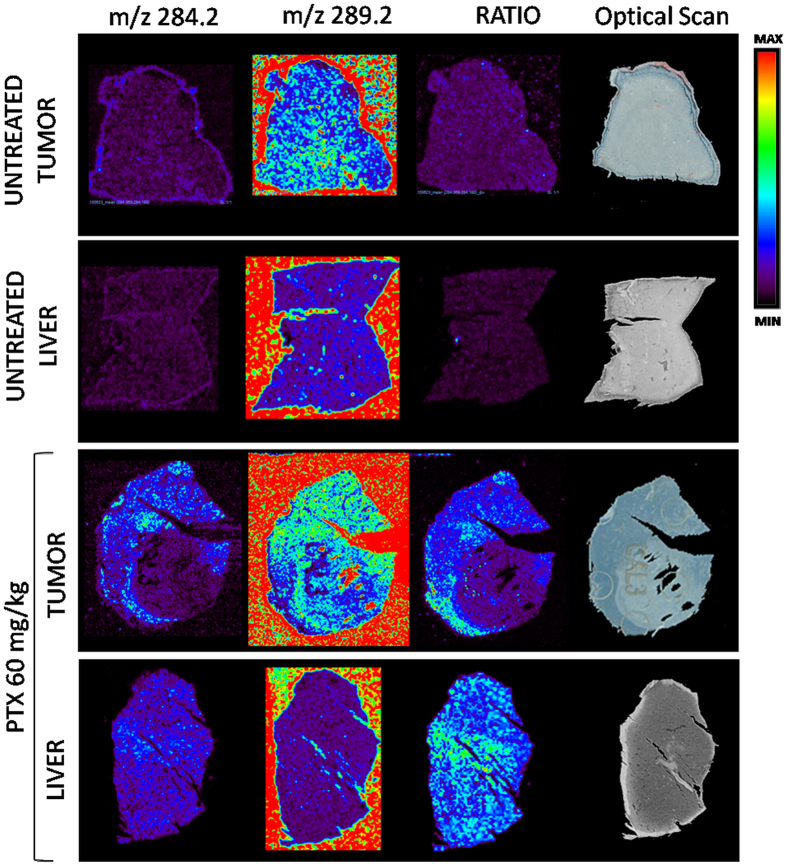
Normalization of PTX signal by internal standard spray approach. Upper panels: untreated tumor and liver sections. Lower panels: tumor and liver sections from a mouse treated with PTX 60 mg/kg i.v. The images of PTX and sprayed d5-PTX ion signal are shown. The third images result from the ratio of the first two images. This normalization approach ensures that the irregular drug distribution seen in the first column is real and not caused by different ion suppression effect in different parts of the tumor. The homogeneous distribution observed in liver, instead, is confirmed by normalization.

**Figure 3:**
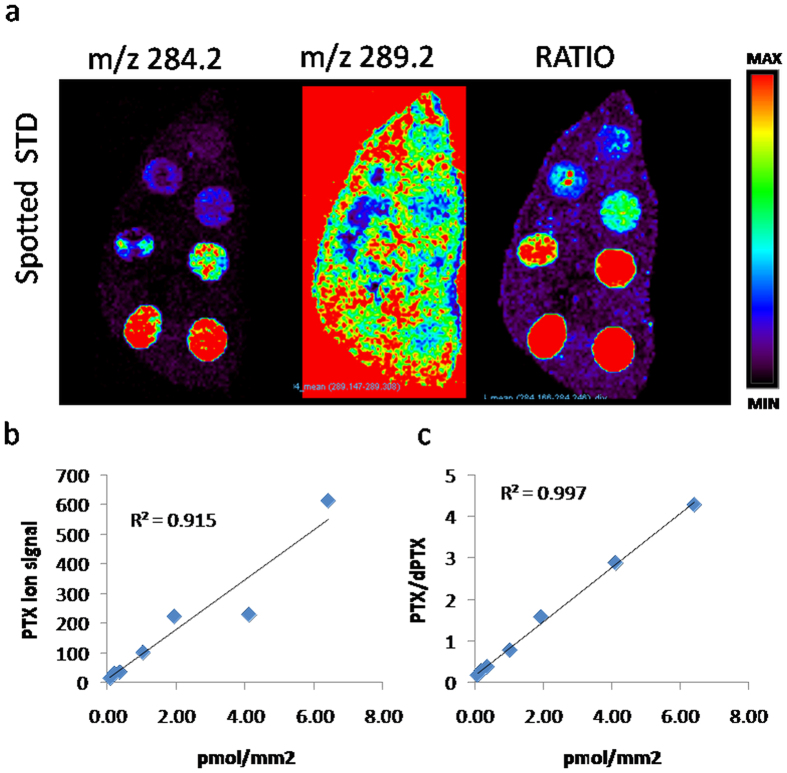
Ion signal linearity. (**a**) PTX spotted on control tissue at increasing concentrations (0.2–15 pmol/spot) showing that ion signal increases linearly with the drug amount. (**b**) Calibration curve. (**c**) Normalized calibration curve: the linearity of the calibration curve is improved by normalization.

A first factor analyzed was how different tissues affected the intensity of the analyte ion signal. The deposition of internal standard (d5-PTX) over sections together with TiO_2_-NP revealed that the different tissue composition and heterogeneity influenced the ion signal intensity. Figure 1 compares the different ion suppression effects originated from various tissues such as liver, spleen and tumors. In particular ion suppression of d5-PTX signal was higher in liver than spleen and tumors. The different ionization of the internal standard is evident even inside the same tumor section highlighting, how much the tumor structural heterogeneity can influence MALDI imaging results. Thus, deposition of the internal standard with the nanoparticles over the tissue is fundamental to compensate ion suppression due to tissue differences or heterogeneity. The normalization of ion signal has been achieved by dividing the ion signal intensity of the drug of interest by the sprayed internal standard in each pixel using dedicated software (Tissue view) (Fig. 2). In tumor sections from a mouse treated with PTX 60 mg/kg i.v. the drug ion signal is clearly detectable while only background noise is visible in untreated tissue; normalization ensures that the irregular drug distribution of PTX in the tumor section is real and not caused by different ion suppression effects in different parts of the tumor. Normalization confirms the homogeneous distribution of the drug in liver section from the treated mouse and balances the higher ion suppression seen in this kind of tissue. Moreover Fig. 3 (panel a) shows that spotting drops of PTX on control tissue at increasing concentrations (0.2–15 pmol/spot), the ion signal increases linearly with the drug amount and that the normalization, based on the sprayed deuterated internal standard, improves the quality of the image, reducing irregularities and variability inside each spot. Even the linearity of the calibration curve built by plotting the ion signal against the spotted PTX amount per surface is improved by normalization enhancing the coefficient of correlation R^2^ from 0.915 to 0.997 (compare panel c and panel b in Fig. 3) confirming what is reported in literature comparing various normalization strategies^18^. In MALDI imaging experiments, when the analytes are desorbed from matrix crystals, the analytes are extracted from the tissue and co-crystallized with the matrix. Extraction efficiencies pose a complex problem as they greatly influence signal intensity. In this case, nanoparticles are homogeneously deposited over the surface in a solvent suspension, and, being a matrix-less approach, do not require co-crystallization^19^. In this case, therefore, homogeneous internal standard deposition over the samples will compensate for the different tissue specific ion suppression effects and laser variability.

**Figure 4:**
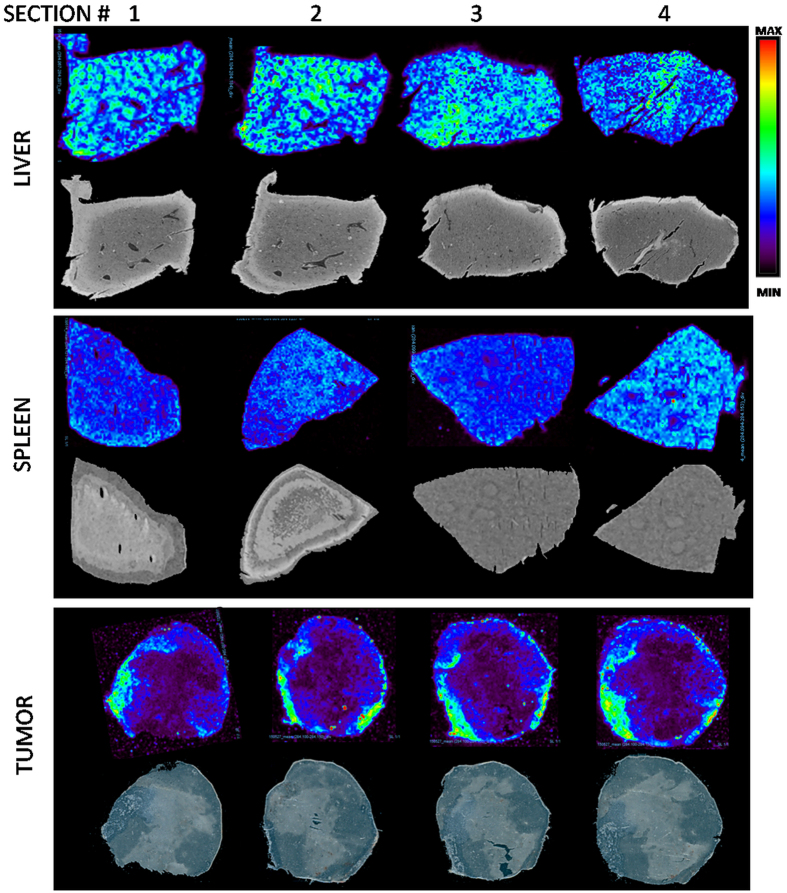
Reproducibility assessment. Normalized images of the PTX distribution in four non-consecutive sections of liver, spleen and tumor. Corresponding optical scans are shown under the MSI images.

The Relative Standard Deviation (RSD%) in ROI drawn on the whole section of different organs, in fact decreases after normalization (Supplementary Figure S1)^20^. Therefore the normalization approach introduced in this method enables to compare PTX distribution in different organs and inside the same tissue or tumor in highly reproducible way. In fact as reported in Fig. 4, we could visualize reliably and consistently PTX distribution in 4 non-adjacent sections (about 300 μm apart) of liver, spleen and tumor. Supplementary Figure S2 shows a second series of sections from liver and spleen explanted from another mouse and from the second half of the same tumor shown in Fig. 4 to further confirm reproducibility. The mean spectrum of the images (Supplementary Figure S3), the mean PTX/d5-PTX ratio and the internal standard ion signal calculated in a ROI including the whole section did not differ comparing the two series of sections for each organ (Supplementary Table S1).

### Paclitaxel distribution in cancer xenografts by MSI

**Figure 5:**
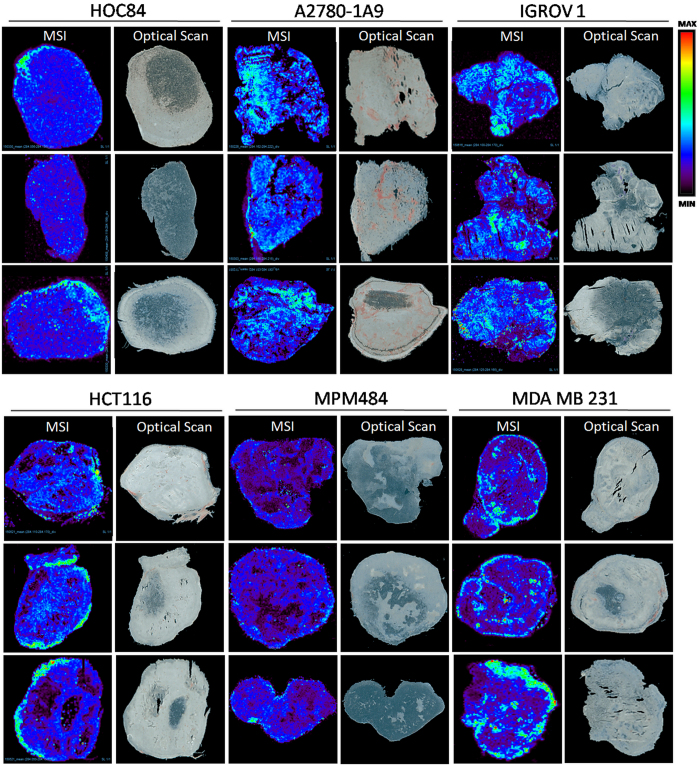
PTX distribution in different cancer models analyzed by MALDI imaging mass spectrometry with corresponding optical scans.

The distribution of PTX was analyzed in five xenograft models with different histological features. The first tumor examined was ovarian xenograft HOC84 which has been characterized in our laboratories for its molecular and histological features and its sensitivity to different chemotherapeutics^21^. In particular HOC84 presents high levels of interstitial matrix, fibrosis but has a good vasculature. We then examined two other ovarian tumor models: A2780-1A9 that is characterized by the presence of disseminated necrotic regions and abnormal irregular vessels, and IGROV1, which is a well vascularized tumor with a characteristic continuous organization of the CD31 positive vessel structures that encircle the tumor complexes growth pattern^12^. The distribution of PTX was also assessed in a tumor xenograft model of colon cancer (HCT116), in a model of breast cancer characterized by the presence of large necrotic areas (MDA-MB-231)^22^ and in a model of malignant pleural mesothelioma with high level of fibrosis (MPM484)^23^ (Fig. 5).

The MSI images show clearly that the distribution of PTX differs among the cancer models with different histopathological characteristics. In particular HOC84 shows the most homogeneous PTX distribution, compared to A2780-1A9, IGROV1 and HCT116 where the drug penetrates unevenly. MPM484 and MDA-MB-231 have the least uniform drug distribution.

The different extent of drug penetration could be ascribed to the histopathological characteristics of the different tumor models. For example it is interesting to notice that large necrotic areas are typical of breast cancer model MDA-MB-231 and their presence correlate with the worst PTX distribution since the drug penetrates scarcely in these regions consistently with what reported in literature by our group in ovarian cancer models^12^. Moreover the drug distribution is really heterogeneous even in malignant pleural mesothelioma model, a highly fibrotic tumor characterized by an extreme resistance to pharmacological therapy^23^.

**Figure 6:**
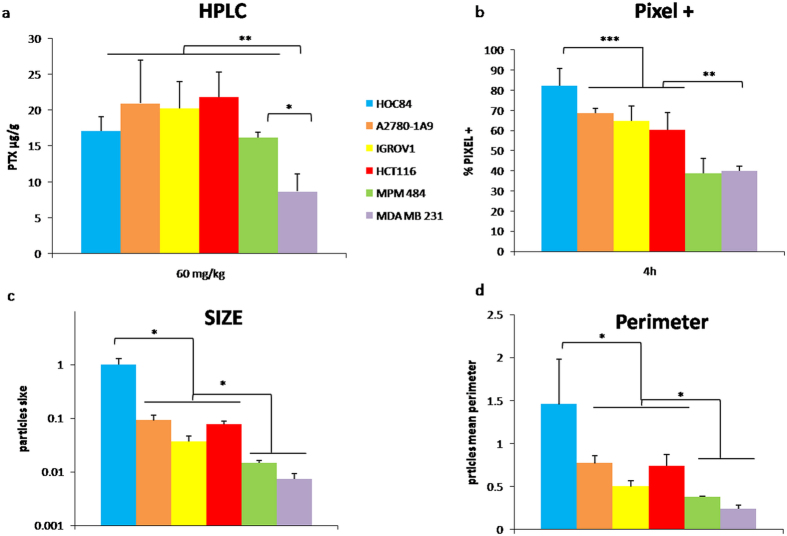
HPLC and Image analysis (**a**) PTX concentrations determined by HPLC analysis in tumor homogenates (mean ± s.d. **One way ANOVA p-value < 0.01; *One way ANOVA p-value < 0.05). (**b**) Percentage of PTX pixels above the threshold (mean ± s.d. **One way ANOVA p-value < 0.01; ***One way ANOVA p-value < 0.001). (**c**) Perimeter and size of the cluster of positive pixels over the threshold in MALDI images (mean ± s.d. *One way ANOVA p-value < 0.05). Blue: HOC84; Orange: A2780-1A9; Yellow: IGROV1; Red: HCT116; Green: MPM484; Violet: MDA-MB-231.

It is interesting to note that the heterogeneous distribution in tumor sections seen in MSI images was not detected by HPLC analysis of tumor homogenates. None of the differences in PTX concentrations in tumor tissues was statistically significant (one way ANOVA with Tukey’s multiple comparisons test p-value > 0.05; HOC84 17.14 ± 1.95 μg/g, n = 5; 1A9 20.98 ± 6.02 μg/g, n = 5; IGROV1 20.30 ± 3.66 μg/g, n = 4; HCT116 21.86 ± 3.45 μg/g, n = 3; MPM484 16.19 ± 3.54 μg/g, n = 3). Only in MDA-MB-231 (8.67 ± 2.48 μg/g, n = 3) the drug concentration was significantly lower (one way ANOVA with Tukey’s multiple comparisons test p-value versus MDA 484 = 0.014) (Fig. 6, panel a).

The different distribution observed by visual inspection in MSI images was confirmed by image analysis. Figure 6 panel b in fact indicates a significantly (one way ANOVA with Tukey’s multiple comparisons test) larger percentage of pixels above the background noise threshold in tumor sections from HOC84 (82.17 ± 3.33%, n = 5) than in sections from A2780-1A9 (68.75 ± 2.34%, n = 5, p-value = 0.0063), IGROV1 (64.83 ± 7.41%, n = 4, p-value = 0.0004) or HCT116 (60.42 ± 6.93%, n = 3, p-value = 0.0002) confirming the more homogeneous PTX tumor distribution. The percentage was even lower in MDA-MB-231 (39.93 ± 1.32%, n = 3, p-value = 0.0016) and in MPM484 (38.81 ± 10.25%, n = 3, p-value = 0.0009).

In addition a more detailed image analysis of the size and shape of the clusters of positive pixels in the MSI images showed that the size and perimeter of aggregates of pixels were higher in the sections from HOC84, than in sections from A2780-1A9, IGROV1 and HCT116. Once again, in MDA-MB-231 and MPM484 the aggregates of positive pixels were the smallest underlying the poor drug distribution (Fig. 6, panel c,d).

**Figure 7:**
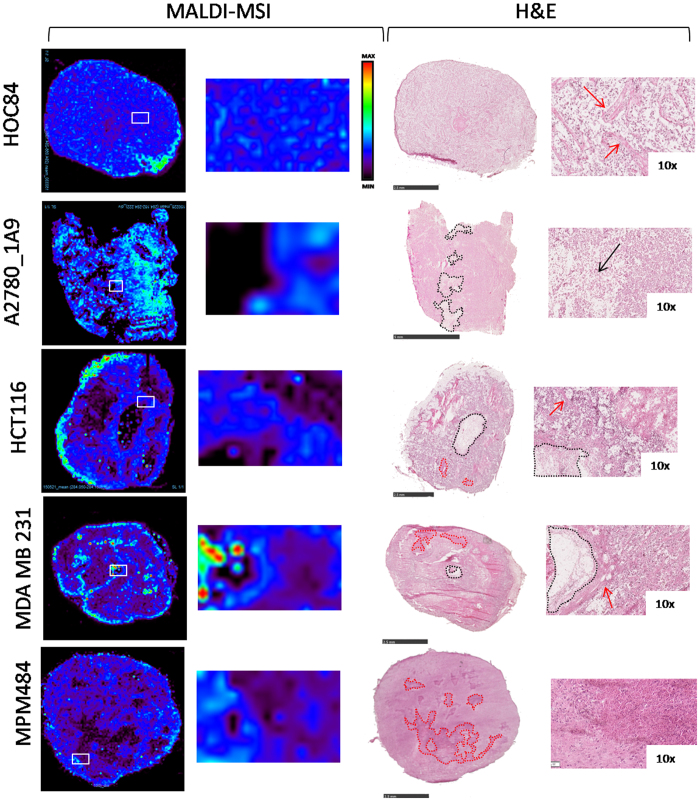
Overlapping of MALDI MSI results and H&E staining. The white squares in MSI images correspond to the region shown in the enlargement. Black dotted lines and arrows in H&E images indicate necrotic regions. Red dotted lines and arrows indicate fibrotic region. H&E scale bar correspond to 2.5 mm except for A2780-1A9 (5 mm).

Figure 7 compares the PTX distribution with histological staining on the adjacent slide. H&E staining of HOC84 tumor slices clearly shows widespread regions of interstitial matrix with evident fibrosis (red arrows), but these do not seem to influence or prevent the drug distribution, at least at macroscopic level. However, in the other models the areas of the tumor where the PTX ion signal is scarce or absent correspond mainly to necrotic (dotted black line and black arrows) and fibrotic regions (dotted red lines and red arrows).

**Figure 8:**
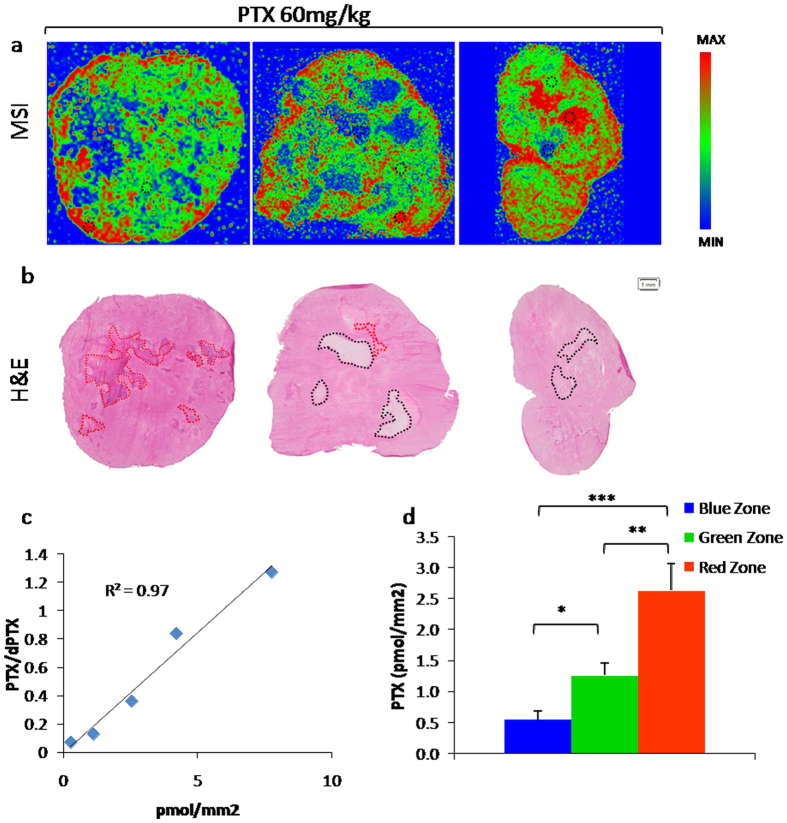
Quantification of PTX in different regions of tumor sections by MALDI MSI in MPM484 xenograft. (**a**) Representative MALDI images in PRISM scale with ROIs (black dotted line) used for quantification (one out of three section analyzed) (**b**) H&E staining of adjacent tumor section, black dotted lines and red dotted lines indicate necrotic or fibrotic regions respectively. (**c**) Calibration curve and (**d**) PTX amount per surface unit in the different color zones (mean ± s.d. **One way ANOVA p-value < 0.01, n = 3, p-value Red versus Green = 0.001, p-value Green versus Blue = 0.0208, p-value Red versus Blue = 0.0001).

Finally, we performed a specific reprocessing of MSI results to verify whether the intra-tumor difference in PTX levels, visible in the tumor sections, was important from a quantitative point of view too. Normalized images of PTX distribution in MPM484 were re-elaborated using a different color scale for quantification with Tissue View. This scale assigns to each pixel a color depending on normalized ion signal intensity following this criterion: red for a high signal (maximum in the section), green for an intermediate signal (approximately from 75% to 25% of maximum) and blue for a low signal (approximately from 25% of maximum to zero). Circular ROIs (25 pixels each) were drawn randomly in representative portions of each color category (3 tumors and 3 sections for each tumor). The PTX amount (pmol/mm^2^) was reverse calculated by interpolation using a calibration curve analyzed in the same experiment. Figure 8 shows the results of quantitative analysis of PTX distribution inside tumor sections by MSI. In the red zone PTX/mm^2^ was respectively 4.76 times and 2.09 times higher than in the blue and green zones. The difference in the PTX amount in the three different zones indicates once again the limits of the quantification approach based on analysis of tumor tissues homogenates as it does not give any information on heterogeneous distribution. Similar results were obtained with MDA-MB 231 and HCT116 (data not shown).

In summary the drug concentrations assessed by traditional analytical method (HPLC) in HOC84, A2780-1A9, IGROV1, HCT116 and MPM484 models were comparable, but MALDI analysis showed that the PTX intratumor distribution is more heterogeneous in the mesothelioma xenograft and more homogeneous in HOC 84 than in the other ovarian models or in HCT116. Instead in MDA-MB-231 model the drug penetration was both scant and irregular.

## Conclusion

An accurate and reproducible imaging method to visualize paclitaxel distribution in tumor tissue is presented. The normalization approach based on sprayed deuterated internal standard compensated of different ion suppression in the various parts of the tumor tissue, reduced experimental variability and avoided artifacts. Analysis of the paclitaxel distribution in different xenograft cancer models showed the wide heterogeneity of the drug distribution inside tumor tissue, highlighting at the same time areas of the tumor where the drug is highly concentrated and areas where it is almost absent. The histological architecture of the tumor sections seemed to influence drug distribution. In fact H&E staining performed on MALDI adjacent sections revealed a correlation between necrotic or fibrotic regions and low level of PTX.

More over this method could be useful in future to evaluate the effects of therapeutic strategies aimed at modifying the tumor microenvironment or altering drug properties to facilitate drug uptake and intratumor distribution.

## Methods

### Drugs and reagents

Paclitaxel (PTX, Indena S.p.A., Milan, Italy) and paclitaxel-d5 (d5-PTX, Toronto Research, Canada) were dissolved in ethanol at a concentration of 1 mg/mL. Serial dilutions of drug were prepared in ethanol 50% from 0.1 to 100 pmol/μL for all MS experiments.

For treatment purposes PTX was dissolved in 50% Cremophor EL (Sigma) and 50% ethanol and further diluted in saline immediately before use.

TiO_2_ nanoparticles (Aeroxide TiO_2_P25, Evonik Industrials, Essen, Germany) were used as a matrix for MSI experiments dissolved at the concentration of 1 mg/mL in ethanol 50%/KCl 0.5%. TiO_2_ nanoparticles suspension was vortexed and sonicated for 3 min just before use, to reduce agglomeration and sedimentation.

### Tumor models

A2780-1A9 is derived from a cell line established from an epithelial-like adenocarcinoma ovarian tumor of an untreated patient. IGROV1-luc human ovarian carcinoma expressing the firefly luciferase gene *luc2* is a variant of human ovarian carcinoma line IGROV1 that is drug resistant and hormone receptor negative^24^. HCT116 is a human colon cancer cell line that has been shown to be invasive and highly motile in *in vitro* studies and highly tumorigenic *in vivo*^25^. Moreover HCT116 results highly sensitive to paclitaxel *in vivo*^26^. HOC84 is a patient-derived xenograft (PDX) model from a high grade serous epithelial ovarian cancer, obtained by serial passages in nude mice^21^. MDA-MB-231 model is derived from a cell line of human triple negative breast carcinoma^22^. This tumor is a well accepted model of estrogen-independent breast cancer tumors, undifferentiated carcinomas containing necrotic areas^27^. MPM484 is a malignant pleural mesothelioma xenograft with epithelioid morphology established in our laboratory from patient-derived cells and maintained by serial passages in nude mice. Its histological and immune-histochemical features reproduce those of the original human tumor^23^.

### Animals and treatments

Six- to eight-week-old female NCr-*Nu/Nu* mice (Envigo, Udine, Italy) were used. HOC84 and MPM484 tumor fragments were implanted subcutaneously in the right flank of nude mice.

A2780-1A9 and HCT116 cells (10 × 10^6^) were injected subcutaneously in the right flank of nude mice. IGROV1-luc cells (1 × 10^6^) were injected orthotopically under the bursa of the mouse ovary, as previously reported by Decio A. *et al*.^24^. MDA-MB-231 cells (10 × 10^6^), co-injected with Matrigel, were inoculated subcutaneously in the right flank of nude mice.

When tumor growth in xenografted mice achieved approximately 300–400 mg (or a determined value of photon count in bioluminescence imaging for orthotopic model) animals were treated either with vehicle (CTRL) or with a single dose of PTX (60 mg/kg i.v.) and euthanized 4 h after treatment. Tumors and organs were explanted then immediately snap-frozen in liquid nitrogen and stored at −80 °C until analysis. The IRFMN adheres to the principles set out in the following laws, regulations, and policies governing the care and use of laboratory animals: Italian Governing Law (D. lgs 26/2014; Authorization n. 19/2008-A issued March 6, 2008 by Ministry of Health); Mario Negri Institutional Regulations and Policies providing internal authorization for persons conducting animal experiments (Quality Management System Certificate–UNI EN ISO 9001:2008 –Reg. N° 6121); the NIH Guide for the Care and Use of Laboratory Animals (2011 edition) and EU directives and guidelines (EEC Council Directive 2010/63/UE). The Statement of Compliance (Assurance) with the Public Health Service (PHS) Policy on Human Care and Use of Laboratory Animals was recently reviewed (9/9/2014) and will expire on September 30, 2019 (Animal Welfare Assurance #A5023-01). The animals were regularly checked by a certified veterinarian responsible for health monitoring, animal welfare supervision, experimental protocols and procedure revision. All surgery was done under general anesthesia, and all efforts were made to minimize suffering. Animal studies were approved by the Mario Negri Institute animal care and use committee (IACUC) and by the Italian Ministerial decree n. 84-2013.

### Mass spectrometry imaging

The paclitaxel distribution in tumors was visualized by MSI according to the method we recently published^11^. Frozen tissues were cut into 10-μm-thick sections using a cryo-microtome (Leica Microsystems, Wetzler, Germany) at −20 °C and mounted on a pre-cooled MALDI plate (Opti-TOF 384 Well insert) by standard thaw-mounting techniques. One section every 300 μm was cut starting from the central part of the tissue. For each section, the adjacent one was mounted on a glass slide for histological analysis (H&E staining) and stored at −20 °C. Three/Five tumors per group and three sections per each tumor were analyzed. The plate was dried in a vacuum drier at room temperature overnight then sprayed with TiO_2_ matrix suspension containing d5-PTX 3 μg/mL, as internal standard, using a BD 180 precision double-action trigger airbrush (FENGDA, Zhejiang, China) with a 0.20 mm nozzle diameter, with nitrogen at 0.2 atm. Different control spots of PTX were applied on the tissue sections, as reference for instrumental calibration or relative quantification.

A MALDI 4800 TOF-TOF (AB SCIEX, Old Connecticut Path, Framingham, MA 01701, USA) was used. It was equipped with a 355-nm Nd:YAG laser with a 200 Hz repetition rate, controlled by 4000 Series Explorer^TM^ software (AB SCIEX). MS spectra were acquired in scan mode with 20 laser shots at an intensity of 6000 arbitrary units, with a bin size of 1.0 ns, in reflectron negative-ion mode. Mass spectra were recorded in full scan profile mode over a limited mass range (m/z 250–300) and this acquisition mode was preferred over the more sensitive selected ion (SIM) or more specific MS/MS mode, in order to have a possible control, over the whole image, of the ion profile, in case of unreasonable signal, and check for possible local problems like instrument calibration or presence of interfering ions.

Images of tissue sections were acquired using the 4800 Imaging Tool software [ www.maldi-msi.org, M. Stoeckli, Novartis Pharma, Basel, Switzerland], with an imaging raster of 100 × 100 μm by plotting the fragment ion at m/z 284.2 corresponding to the side chain with the amide-acyl group of PTX.

Tissue View software 1.1 (AB SCIEX) was used to process and display the ion distribution inside the tumor sections. A classical RAINBOW color scale was used to create images of drug distribution. For quantitative analysis the acquired images were re-elaborated using PRISM color scale, in order to highlight three levels of ion signal intensity.

The distribution of PTX ion signal in the normalized images was analyzed by the software ImageJ (imagej.nih.gov/ij). Regions of interest (ROIs) enclosing the whole tumor section were drawn. We defined a threshold for enhancing pixels as a signal change greater than the background noise in untreated samples. The number of pixels that exceeded this threshold was calculated and divided by the total number of pixels in the ROIs to obtain the percentage of pixels where the drug is present^28^. In addition, we did a “particles analysis” on images after applying the threshold. This measures the characteristics (number, mean size, mean perimeter or circularity) of clusters formed by adjacent positive pixels (above the threshold) in the image in order to describe the drug distribution in detail.

### Quantification of paclitaxel

The total concentration of PTX in tumors was determined by HPLC as previously described^29^. Tissues were homogenized in 0.2 M CH_3_COONH_4_ pH 4.5 (1:2 wt/vol) and 0.5 mL of homogenate tissues for each study sample was assayed together with a five-points standard calibration curve prepared in the corresponding control tissues obtained from untreated mice at concentrations ranging from 0.1 to 5 μg/sample. The limit of quantification (LOQ) was 0.08 μg/sample.

### Statistical analysis

Statistical analysis was performed with GraphPad Prism version 6.01 software (GraphPad software, Inc., La Jolla, CA, USA). One way ANOVA with Tukey’s multiple comparisons test was performed to compare drug content and distribution in the different tumor models analyzed. Student’s t test was performed to evaluate differences in RSD% with or without normalization. P-value < 0.05 was considered significant.

## Additional Information

**How to cite this article**: Giordano, S. *et al*. Heterogeneity of paclitaxel distribution in different tumor models assessed by MALDI mass spectrometry imaging. *Sci. Rep.* **6**, 39284; doi: 10.1038/srep39284 (2016).

**Publisher's note:** Springer Nature remains neutral with regard to jurisdictional claims in published maps and institutional affiliations.

## Supplementary Material

Supplementary Information
