# Supplementary material for: Heterogeneity of paclitaxel distribution in different tumor models assessed by MALDI mass spectrometry imaging
Source: Sci Rep. 2016 Dec 21;6:39284. doi: 10.1038/srep39284 (PMC5175283; doi:10.1038/srep39284)
Supplement: Supplementary Information [file srep39284-s1.pdf]

Heterogeneity of paclitaxel distribution in different tumor models assessed by MALDI mass spectrometry imaging

Giordano Silvia[2], Zucchetti Massimo[1], Decio Alessandra[1], Cesca Marta[1], Fuso Nerini Ilaria[1], Maiezza Marika[1], Ferrari Mariella[1], Licandro Simonetta Andrea[1], Frapolli Roberta[1], Giavazzi Raffaella[1], D'Incalci Maurizio[1], Davoli Enrico[2], Morosi Lavinia[1].

1 Department of Oncology, IRCCS Istituto di Ricerche Farmacologiche Mario Negri, Via La Masa, 19 – 20156 Milano, Italy
2 Mass Spectrometry Laboratory, IRCCS Istituto di Ricerche Farmacologiche Mario Negri, Via La Masa, 19 – 20156 Milano, Italy

Corresponding author: Morosi Lavinia

Content:

- The normalization with internal standard approach increases reproducibility reducing signal variability due to inhomogeneous matrix deposition, analyte extraction or laser shot to shot variability. RSD % in ROI drawn on the entire section of different organs, in fact decrease in a statistically significant way after normalization (*** student t test p-value < 0.001) **Figure S-1**

- A second series of normalized images of the PTX distribution in four non-consecutive sections of liver, spleen and tumor. Corresponding optical scans are shown under the MSI images**. Figure S-2**

- The reproducibility of the MSI method to visualize paclitaxel was confirmed by the comparison of the mean spectra calculated in a ROI including the whole section of 4 non adjacent sections (about 300µm between sections) of liver, spleen and tumor. **Figure S-3**

- The mean PTX/d5-PTX ratio, the standard deviation (SD) and the internal standard ion signal calculated in a ROI including the whole section do not vary in a significant way comparing the two series of sections for each organ analyzed **Table S-1**

- Quantitative analysis of PTX distribution inside tumor sections by MSI. PTX/mm^2 was calculated from PTX/d5PTX ratio in three randomly selected ROIs for each color band in three sections for each tumor (3 tumors) respectively 4.76 times and 2.09 times higher than in the blue and green zones.

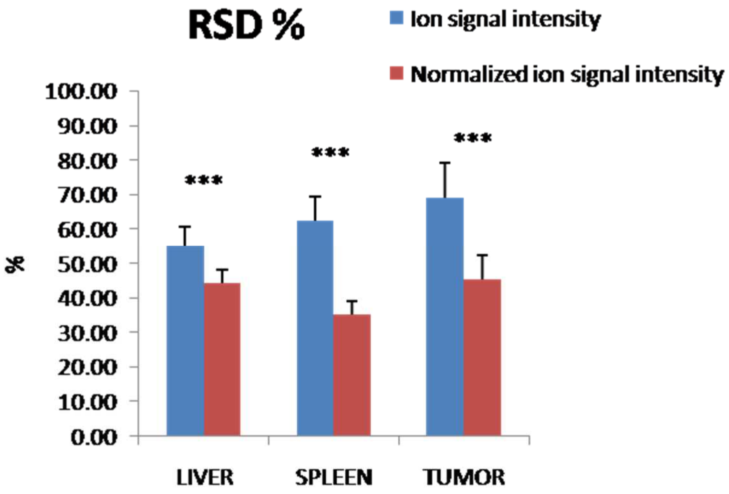
RSD %
Ion signal intensity
Normalized ion signal intensity
100.00
90.00
80.00
70.00
60.00
50.00
40.00
30.00
20.00
10.00
0.00
%
***
***
***
LIVER
SPLEEN
TUMOR

Figure S-1

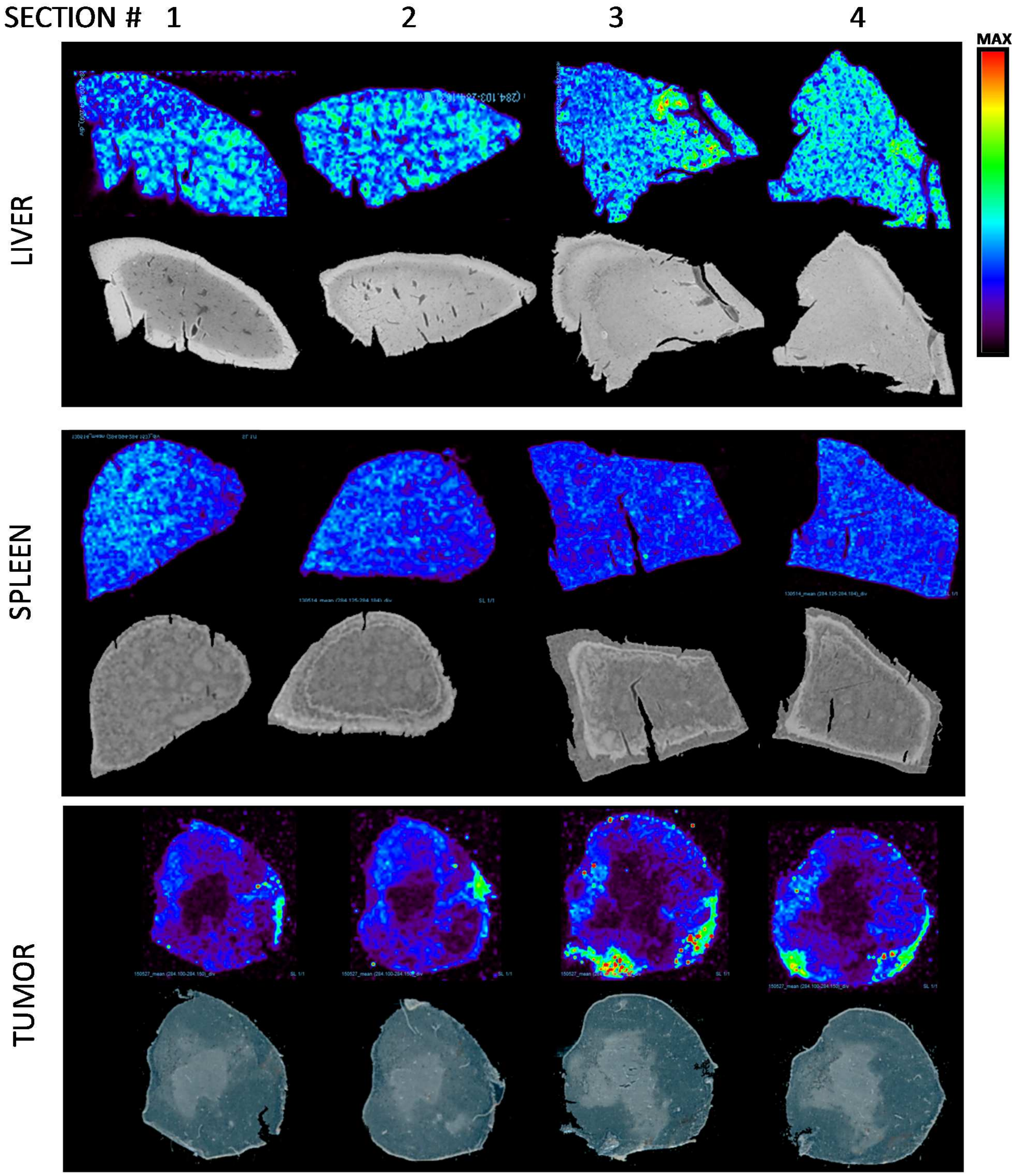

Figure S-2

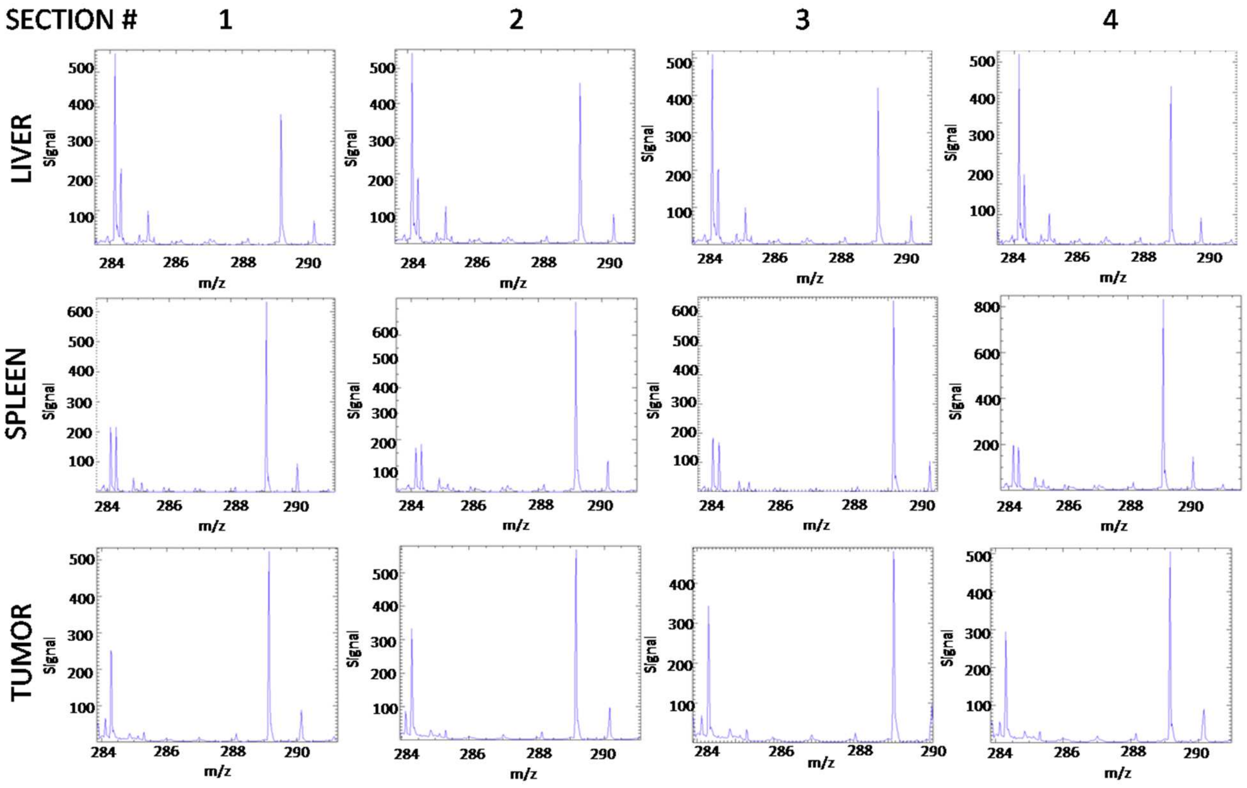

Figure S-3

Table S-1

		Ratio PTX/dPTX	m/z 289.2
**Organ**	**Section #**	**MEAN**	**MEAN**
**LIVER A**	**1**	2.049	233.580
	**2**	1.394	255.650
	**3**	1.247	271.460
	**4**	1.575	284.880
**LIVER B**	**1**	1.862	204.175
	**2**	2.316	259.746
	**3**	1.822	206.164
	**4**	1.677	233.654
**p value test T**		0.164	0.084
**SPLEEN A**	**1**	0.251	288.740
	**2**	0.310	366.199
	**3**	0.319	309.298
	**4**	0.387	370.474
**SPLEEN B**	**1**	0.310	308.531
	**2**	0.378	342.738
	**3**	0.400	243.965
	**4**	0.482	296.948
**p value test T**		0.143	0.264
**TUMOR A**	**1**	0.187	262.682
	**2**	0.158	311.040
	**3**	0.184	276.485
	**4**	0.187	255.133
**TUMOR B**	**1**	0.200	282.242
	**2**	0.179	319.642
	**3**	0.203	318.551
	**4**	0.169	230.429
**p value test T**		0.468	0.657

Table S-2

	Tumor	Section #	PTX/dPTX	pmol/mm2	MEAN	DEV ST	MEAN	DEV ST
BLUE	A	1	0.089	0.559	0.476	0.072	0.552	0.135
		2	0.069	0.441				
		3	0.067	0.429				
	B	1	0.065	0.418	0.473	0.049		
		2	0.081	0.512				
		3	0.077	0.488				
	C	1	0.121	0.747	0.708	0.244		
		2	0.152	0.929				
		3	0.07	0.447				
GREEN	A	1	0.196	1.188	1.218	0.164	1.261	0.194
		2	0.176	1.071				
		3	0.231	1.394				
	B	1	0.182	1.106	1.092	0.063		
		2	0.168	1.024				
		3	0.189	1.147				
	C	1	0.329	1.971	1.473	0.431		
		2	0.201	1.218				
		3	0.203	1.229				
RED	A	1	0.412	2.459	2.722	0.265	2.631	0.436
		2	0.456	2.718				
		3	0.502	2.988				
	B	1	0.307	1.841	2.157	0.497		
		2	0.317	1.900				
		3	0.458	2.729				
	C	1	0.549	3.265	3.014	0.242		
		2	0.467	2.782				
		3	0.503	2.994				
